# Exercise, Nutrition and the Brain

**DOI:** 10.1007/s40279-014-0150-5

**Published:** 2014-05-03

**Authors:** Romain Meeusen

**Affiliations:** Department of Human Physiology, Vrije Universiteit Brussel, Pleinlaan 2, 1050 Brussels, Belgium

## Abstract

Accumulating evidence suggests that diet and lifestyle can play an important role in delaying the onset or halting the progression of age-related health disorders and can improve cognitive function. Exercise has been promoted as a possible prevention for neurodegenerative diseases. Exercise will have a positive influence on cognition and it increases the brain-derived neurotrophic factor, an essential neurotrophin. Several dietary components have been identified as having effects on cognitive abilities. In particular, polyphenols have been reported to exert their neuroprotective actions through the potential to protect neurons against injury induced by neurotoxins, an ability to suppress neuroinflammation, and the potential to promote memory, learning, and cognitive function. Dietary factors can affect multiple brain processes by regulating neurotransmitter pathways, synaptic transmission, membrane fluidity, and signal-transduction pathways. Flavonols are part of the flavonoid family that is found in various fruits, cocoa, wine, tea and beans. Although the antioxidant effects of flavonols are well established in vitro, there is general agreement that flavonols have more complex actions in vivo. Several cross-sectional and longitudinal studies have shown that a higher intake of flavonoids from food may be associated with a better cognitive evolution. Whether this reflects a causal association remains to be elucidated. Several studies have tried to ‘manipulate’ the brain in order to postpone central fatigue. Most studies have clearly shown that in normal environmental circumstances these interventions are not easy to perform. There is accumulating evidence that rinsing the mouth with a carbohydrate solution will improve endurance performance. There is a need for additional well controlled studies to explore the possible impact of diet and nutrition on brain functioning.

## Introduction

Physical activity has been associated with the reduction of a number of physical and mental disorders. There is now ample evidence that physical activity decreases the incidence of cardiovascular disease, colon and breast cancer, and obesity, but also diseases such as Alzheimer’s, depression, and anxiety. Nutrition has classically been perceived as a means to provide energy and building materials to the body. However, its ability to prevent and protect against diseases is starting to be recognized. Nutrition and exercise are therefore used as interventions to reverse these possible negative health effects. Recent data indicate that not only general health, but also brain functioning, is influenced through exercise and nutritional interventions [1]. This article will describe how exercise and nutrition can influence brain development, (brain) performance and cognition.

## Brain Development, Exercise, Nutrition and Cognition

There are strong indications that children are growing increasingly sedentary and unfit, and that these lifestyle factors are related to an earlier onset of several chronic diseases such as type 2 diabetes and obesity. Several cross-sectional and longitudinal studies have given proof of an association between being overweight and poor academic performance [2, 3]. Aerobic fitness has also been linked with cognition and academic achievement [2]. Several studies have suggested that children’s cognitive ability and school performance may be affected by their general physical condition [2, 3].

Nutrition can also substantially influence the development and health of brain structure and function. Nutrition provides the proper building blocks for the brain to create and maintain connections, which is critical for improved cognition and academic performance. Dietary factors have a broad and positive action on neuronal function and plasticity. For example, the omega-3 fatty acids provide building material to the brain. They are essential for supporting intercellular signaling events, and therefore positively influence synaptic function. However, diets rich in sugar, saturated fats, or high in calories are considered deleterious for neural function, as they act to elevate levels of oxidative stress and to reduce synaptic plasticity and cognitive functions [4]. Brain function is certainly dependent on adequate nutrition, and short-term variations in the amount and composition of nutrient intake in healthy individuals influence measures of cognitive function. Studies have shown that eating breakfast is associated with several positive effects on the cognitive functioning of well-nourished children [3, 5]. Exercise has been shown to interact with dietary interventions—increasing the positive effects on brain functioning, and decreasing the unhealthy effects of a high-fat diet. The overall evidence seems to indicate that combined strategies based on exercise and dietary management can derive maximal benefit for neural health promotion [5]. Furthermore, Pivik et al. [6] recently determined the influence of a morning meal on complex mental functions in children (aged 8–11 years). Brain activity was measured by electroencephalography while children solved simple addition problems after an overnight fast and again after having eaten or skipped breakfast. Fed children showed a significant increase in correct responses, while children who continued to fast did not. Taken together, the findings suggest that neural network activity involved in processing numerical information is functionally enhanced and performance is improved in children who have eaten breakfast, whereas greater mental effort is required for this mathematical thinking in children who skip breakfast [6].

Evidence from cross-sectional studies has consistently shown linear age-related declines in cognitive functions such as processing speed, short-term memory, working memory, and long-term memory [7]. The age-related decrements in cognition have been associated with changes in brain structure and function, and physical activity might play a central role in ameliorating age-associated cognitive losses [7]. Recent meta-analyses [8, 9] on the effects of physical activity on human cognitive aging have shown that aerobic exercise had general and selective effects that were beneficial to cognitive function in older adults. These findings suggest that although cognitive performance declines in a global and linear fashion with age, physical activity and aerobic fitness may serve to protect against age-related loss of cognitive function, with the greatest benefits derived for processes requiring extensive amounts of executive control.

## Brain Structure

It seems that these findings are also translated into structural changes in the brain. A recent study showed that the structure of the brain, specifically the volume of the hippocampus (a brain area very important in learning and memory), is greater in physically fit children when compared with age-matched non-physically fit children [10]. Another study reported several additional brain regions that are structurally different based on the child’s level of physical fitness [11]. The dorsal striatum, believed to play a role in cognitive control and inhibition, was larger in those children who were more physically fit [11]. It is possible that the observed structural differences between physically fit and unfit children may partly underlie the foundation for the functional brain differences seen in obese children compared with healthy weight children.

Brain morphology responds to specific stimuli during the lifespan. Brain plasticity exists not only in children, but at all ages. Raji et al. [12] used functional magnetic resonance imaging to assess gray and white matter volume atrophy in 94 elderly adults (mean age of 77 years). Results showed that body mass index, fasting plasma insulin, and type 2 diabetes were strongly associated with atrophy of the frontal, temporal, and subcortical regions of the brain. These data indicate that being overweight and obese may be associated with marked decreases in brain volume, and provide a greater understanding of the underlying causes of obesity-related changes in cognitive dysfunction. Given that several of the brain regions demonstrating decreases in volume are associated with attention, memory, and the control of cognition, obesity-related deficits in cognitive and scholastic performance might be expected to be mediated by these brain regions.

Exercise training can still influence brain morphology at older ages. Hippocampal and medial temporal lobe volumes are larger in highly fit adults, and physical activity training increases hippocampal perfusion. Erickson et al. [13] showed, in a 1-year randomized controlled trial with 120 older adults (aged 55–80 years), that aerobic exercise training increased the size of the anterior hippocampus, leading to improvements in spatial memory. Exercise training increased hippocampal volume by 2 %, effectively reversing age-related losses in volume by 1–2 years. Hippocampal volume declined in the control group. Caudate nucleus and thalamus volumes were unaffected by the intervention. These findings indicate that aerobic exercise training is effective at reversing hippocampal volume loss in older adults, which is accompanied by improved memory function [13].

## Mechanisms

Animal research has shown that enriched environments, including access to running wheels, has a positive effect on neuronal growth and on the neural systems that are involved in learning and memory. This neuroplasticity refers to the ability of the brain to adapt to environmental change, respond to injury, and to acquire novel information by modifying neural connectivity and function. Neurotrophins support neuroplasticity, and they are capable of signaling neurons to survive, differentiate, or grow. Neurotrophic factors not only play a role in neurobiology, but also in central and peripheral energy metabolism [14]. Their effect on synaptic plasticity in the central nervous system (CNS) involves elements of cellular energy metabolism. Acute exercise and training seem to be key interventions to trigger the processes through which neurotrophins mediate energy metabolism and neural plasticity. Of all neurotrophins, brain-derived neurotrophic factor (BDNF) seems to be the most susceptible to regulation by exercise and physical activity [14]. BDNF has a wide repertoire of neurotrophic and neuroprotective properties in the CNS and the periphery. These include neuronal protection and survival, neurite expression, axonal and dendritic growth and remodeling, neuronal differentiation, and synaptic plasticity such as synaptogenesis in arborizing axon terminals, and synaptic transmission efficacy. Animal studies have also revealed a neuroendocrine and/or metabotrophic capacity of BDNF in the periphery [14]. BDNF reduces food intake, increases oxidation of glucose, lowers blood glucose levels, and increases insulin sensitivity. In animals, a high-fat diet reduces the hippocampal concentration of BDNF, but exercise is able to reverse this dietary decrease [15]. Furthermore, in mice it has been shown that there is a central interaction between the adipocyte-derived hormone leptin, which plays a key role in regulating appetite and energy metabolism, and BDNF expression in the hypothalamus [16]. Araya et al. [17] found that serum BDNF increases in insulin-resistant, overweight and obese individuals after a reduced energy diet. These findings confirm that BDNF is not only essential in the neuronal system, but is intimately connected with central and peripheral molecular processes of energy metabolism and homeostasis [18].

**Fig. 1:**
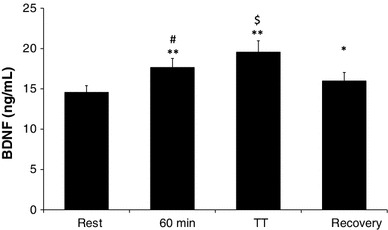
Serum BDNF concentration (ng/mL, mean ± SD) at rest, after 60 min of cycling at 55 % of maximum wattage, after a TT and following 15 min of recovery, performed in an environmental temperature of 18 °C under placebo treatment (*n* = 22). Reproduced with modification from Goekint [26], with permission. *Significantly different from rest (*p* < 0.05); **significantly different from rest (*p* < 0.01); ^#^significantly different from rest (*p* < 0.01) and recovery (*p* < 0.05); ^$^significantly different from rest, 60 min and recovery (*p* < 0.01). *BDNF* brain-derived neurotrophic factor, *SD* standard deviation, *TT* time trial

In the search of mechanisms underlying plasticity and brain health, exercise is known to induce a cascade of molecular and cellular processes that support (brain) plasticity. BDNF could play a crucial role in these mechanisms. Therefore, since the early 1990s, studies have begun to investigate the effects of physical activity and acute exercise and/or training on the concentrations of BDNF, first in animals [19, 20] and then in humans [14, 21]. It was shown in a series of studies that exercise, independent of the central neurotransmitter system, increased BDNF release [22–26] (Fig. 1). After training for 8 weeks, baseline BDNF levels are lower, possibly due to a receptor adaptation, while detraining will abolish the exercise-induced effects [24]. Little is known about the effect of resistance exercise on hippocampus-dependent memory, although this type of exercise is increasingly recommended to improve muscle strength and bone density and to prevent age-related disabilities. It was shown that resistance training does not significantly increase peripheral BDNF levels [22]; however, it seems that resistance exercise increases cognitive performance, especially in the elderly population [27]. To explore the possible underlying mechanisms, Cassilhas et al. [28] performed a study in which animals underwent an aerobic training program or a resistance training program for 8 weeks. Trained animals showed an increased cognition compared with control animals on a water maze test. It was shown that different underlying mechanisms were responsible for this cognitive improvement. In endurance-trained rats there was a significant increase in brain BDNF as well as its tyrosine kinase B receptor. However, in the resistance-trained animals, BDNF did not increase but there was a significant increase of insulin such as insulin growth factor 1 and its receptor. Further analysis showed that both training regimens induced an increase in the expression of synapsin 1 and synatophysin, triggering the hypothesis that both aerobic and resistance exercise can employ divergent molecular mechanisms, but achieve similar results on learning and spatial memory.

## Nutrition and Cognition

The brain is a very metabolically active organ accounting for a high percentage of the total metabolic rate. As well as affecting the architecture of the brain, nutrition can also potentially influence functioning from moment to moment [29]. Non-invasive imaging techniques have clearly demonstrated that simply thinking about food can modulate neural activity in specific brain areas known to be involved in the cognitive controls of appetitive behaviors, and can lead to physiological responses such as saliva, gastric acid, and insulin secretion [30].

There has recently been growing interest, supported by a number of epidemiological and experimental studies, on the possible beneficial effects of polyphenols on brain health [31, 32]. Polyphenols are abundant micronutrients in plant-derived foods and are powerful antioxidants. Fruits and beverages such as tea, red wine, cocoa, and coffee are major dietary sources of polyphenols. Polyphenols have been reported to exert their neuroprotective actions through the potential to protect neurons against injury induced by neurotoxins, an ability to suppress neuroinflammation, and the potential to promote memory, learning, and cognitive function [31]. Despite significant advances in understanding the biology of polyphenols, they are still mistakenly regarded as simply acting as antioxidants. However, recent evidence suggests that their beneficial effects involve decreases in oxidative/inflammatory stress signaling, increases in protective signaling and neurohormetic effects, leading to the expression of genes that encode antioxidant enzymes, neurotrophic factors, and cytoprotective proteins [32].

The largest group of polyphenols is the flavonoids. There are six dietary groups of flavonoids: flavones (e.g. apigenin, luteolin), which are found in parsley and celery; flavanones/flavanonols (e.g. hesperetin, naringenin/astilbin, engeletin), which are mainly found in citrus fruit, herbs (oregano), and wine; isoflavones (e.g. daidzein, genistein), which are mainly found in soy and soy products; flavonols (e.g. kaempferol, quercetin), which are found in onions, leeks, and broccoli; flavanols [e.g. (+)-catechin, (−)-epicatechin, epigallocatechin, and epigallocatechin gallate], which are abundant in green tea, red wine, and chocolate; anthocyanidins (e.g. pelargonidin, cyanidin, andmalvidin), whose sources include red wine and berry fruits. The non-flavonoid group of polyphenols may be separated into two different classes: the phenolic acids, including the hydroxybenzoic acids (C1–C3 skeleton) and hydroxycinnamic acids (C3–C6 skeleton), and the stilbenes (C6–C2–C6 skeleton). Caffeic acid is generally the most abundant phenolic acid, and is mainly found as the quinic ester, chlorogenic acid, in blueberries, kiwis, plums, and apples. Resveratrol, the main stilbene, can be found in the cis or trans configurations, either glucosylated (piceid) or in lower concentrations as the parent molecule of a family of polymers such as viniferins, pallidol, or ampelopsin A. Resveratrol dietary sources include grapes, wine, and peanuts [32].

Polyphenols have been associated with a reduced risk of developing dementia, an improved cognitive performance in normal aging and an improved cognitive evolution [32]. Letenneur et al. [33] performed a prospective cohort study over a 10-year period among subjects aged 65 years or older to investigate the relation among antioxidants, cognitive decline, and dementia. A total of 1,640 subjects free from dementia at baseline in 1990 and with reliable dietary assessments were re-examined four times over a 10-year period. Cognitive functioning was assessed through three psychometric tests. Information on flavonoid intake was collected at baseline. After adjustment for age, sex, and educational level, flavonoid intake was associated with better cognitive performance at baseline and with a better evolution of the performance over time. Subjects included in the two highest quartiles of flavonoid intake had better cognitive evolution than subjects in the lowest quartile. After 10 years’ follow-up, subjects with the lowest flavonoid intake had significantly worse performance on psychometric tests, even after adjustment for several other potential confounders. In a cross-sectional study, Nurk et al. [34] examined the relation between the intake of three common foodstuffs that contain flavonoids (chocolate, wine, and tea) and cognitive performance. More than 2,000 participants (aged 70–74 years; 55 % women) recruited from the population-based Hordaland Health Study in Norway underwent cognitive testing. Participants who consumed chocolate, wine, or tea had significantly better mean test scores and a lower prevalence of poor cognitive performance than those who did not. Participants who consumed all three studied items had the best test scores and the lowest risks of poor test performance. The associations between the intake of these foodstuffs and cognition were dose dependent, with maximum effect at intakes of 10 g/day for chocolate and 75–100 mL/day for wine, but were approximately linear for tea. Most cognitive functions tested were influenced by the intake of these three foodstuffs. The effect was most pronounced for wine and modestly weaker for chocolate intake. Therefore, in the elderly, a diet high in some flavonoid-rich foods is associated with better performance in several cognitive abilities in a dose-dependent manner.

The neuroprotective actions of dietary polyphenols involve a number of effects within the brain, including a potential to protect neurons against injury induced by neurotoxins, an ability to suppress neuroinflammation, and the potential to promote memory, learning, and cognitive function. While many of the mechanisms underpinning their beneficial effects remain to be elucidated, it has become clear that they partly involve decreases in oxidative/inflammatory stress signaling, increases in protective signaling, and may also involve hormetic effects to protect neurons against oxidative and inflammatory stressors.

Emerging evidence suggests that dietary-derived flavonoids have the potential to improve human memory and neurocognitive performance by their ability to protect vulnerable neurons, enhance existing neuronal function, and stimulate neuronal regeneration [32]. Long-term potentiation (LTP) is widely considered to be one of the major mechanisms underlying memory acquisition, consolidation and storage in the brain, and is known to be controlled at the molecular level by the activation of a number of neuronal signaling pathways. These pathways include the phosphatidylinositol-3 kinase/protein kinase B/Akt (Akt), protein kinase C, protein kinase A, calcium–calmodulin kinase and mitogen-activated protein kinase pathways. Growing evidence suggests that flavonoids exert effects on LTP, and consequently memory and cognitive performance, through their interactions with these signaling pathways [35]. Of particular interest is the ability of flavonoids to activate the extracellular signal-regulated kinase and the Akt signaling pathways, leading to the activation of the cyclic adenosine monophosphate response element binding protein, a transcription factor that increases the expression of a number of neurotrophins important in LTP and long-term memory. One such neurotrophin is BDNF, which is known to be crucial in controlling synapse growth, promoting an increase in dendritic spine density, and enhancing synaptic receptor density [35].

While at present the balance of evidence does suggest that polyphenol effects contribute to the benefits of a high intake of fruits and vegetables, the extent of their contribution in vivo and at physiologically relevant concentrations remains uncertain. The limited bioavailability of these substances is often overlooked, particularly in some animal and in vitro work. Clearly, this may limit efficacy in some supplementation studies. More work needs to be done to prove whether this class of compounds is most likely to result in health benefits and to determine their beneficial effects in slowly developing neurodegenerative disorders. In view of their multiple biological activities, the consumption of polyphenol-rich foods throughout life holds the potential to limit neurodegeneration and to prevent or reverse age-dependent deteriorations in cognitive performance. However, the therapeutic and pharmacological potential of these natural compounds still remains to be translated to humans in clinical conditions [35].

## Nutrition and Fatigue

Nutritional interventions can be used not only to influence cognition but also to manipulate fatigue. There has been an extensive amount of research on the effects of nutritional manipulations on exercise performance, especially trying to postpone ‘central fatigue’. Fatigue can be defined as an acute impairment of exercise performance, which leads to an inability to produce maximal force output, possibly due to metabolite accumulation or substrate depletion [36]. It includes both an increase in the perceived effort necessary to exert a desired force or power output, and the eventual inability to produce that force or power output [37]. Fatigue not only occurs at the peripheral level, as there is ample evidence that mechanisms in the CNS are also implicated in the genesis of fatigue.

Brain neurotransmitters and especially the central monoamines are strong candidates for inducing the centrally mediated effects of fatigue during exercise. The monoamines serotonin (5-HT), dopamine, and noradrenaline play a key role in signal transduction between neurons, and exercise-induced changes in the concentrations of these neurotransmitters (especially 5-HT and dopamine) have been linked to central fatigue. After the initial work by Acworth et al. [38], Newsholme et al. [39] developed the first hypothesis implicating changes in central neurotransmission to explain fatigue, i.e. the ‘central fatigue hypothesis’. This hypothesis was based on disturbances in brain 5-HT concentrations, as this neurotransmitter is involved in changes in sleep–wakefulness, emotion, sleep, appetite, the hypothalamic–pituitary axis, and numerous physiological functions [40]. During exercise, the entry of tryptophan (a precursor of 5-HT) into the CNS through the blood–brain barrier is favored by increased muscle use of branched-chain amino acids (BCAAs) and elevated plasma fatty acids, as this elevates the ratio of unbound tryptophan to BCAA. This increases the amount of tryptophan crossing the blood–brain barrier, consequently leading to higher 5-HT concentrations in the brain [40–42]. Events arising entirely from within the brain can influence an individual’s sensation of fatigue and thus potentially affect performance. This opens an opportunity to manipulate the CNS through changes in diet or supplementation with specific nutrients, including amino acids (BCAAs, tyrosine), carbohydrate, and caffeine.

## Use of Nutrition to Influence Central Fatigue

A number of studies have attempted to postpone ‘central fatigue’ through nutritional interventions. Water intake and several amino acids, carbohydrate, caffeine, and other foodstuffs have been examined. It seems that not all studies have been conclusive, indicating that several aspects of the effects of nutrition on ‘central fatigue’ remain to be explored.

### Hydration Status

The effect of hydration status on the performance of various exercise tasks has been extensively studied, but hydration status can also affect the brain. There is some limited evidence that dehydration results in a change in brain volume [43], but this response has not been demonstrated in all studies [44]. Dehydration [45] and hyperthermia [46] also appear to result in transient opening of the blood–brain barrier, and this may have implications for the stability of the cerebral environment during exercise. It seems that healthy volunteers exhibit cognitive compensating mechanisms for increased tiredness and reduced alertness during slowly progressive moderate dehydration. In a study using magnetic resonance imaging, Kempton et al. [47] showed that when dehydrated, subjects exerted a higher level of neuronal activity in order to achieve the same performance level. Given the limited availability of brain metabolic resources, these findings suggest that prolonged states of reduced water intake may adversely impact executive functions such as planning and visuospatial processing [47]. This might have implications in team sports and those sports in which ‘decision making’ is important.

### Branched-Chain Amino Acids

Fernstrom [48] clearly indicated the importance of the BCAAs leucine, isoleucine, and valine. They participate directly and indirectly in a variety of important biochemical functions in the brain, such as protein synthesis, energy production, and the synthesis of 5-HT, dopamine, and noradrenaline, which are derived from the aromatic amino acids tryptophan, phenylalanine, and tyrosine [48]. The ingestion of BCAAs causes a rapid elevation of their plasma concentrations and increases their uptake into the brain. It was hypothesized that by reducing the production of 5-HT in the brain, feelings of fatigue could be attenuated and performance enhanced. Supplementation of BCAAs has been proposed as a possible strategy to limit the development of central fatigue. Although this is a very attractive theory, there is limited or only circumstantial evidence to suggest that exercise performance in humans can be altered by nutritional manipulation with BCAA supplements. Madsen et al. [49], Strüder et al. [50], and van Hall et al. [51] attempted to influence the plasma-free tryptophan to BCAA ratio with BCAA supplementation, but failed during exercise in normal ambient temperatures [52]. Ingestion of a BCAA solution before and during prolonged exercise in glycogen-depleted individuals did not influence exercise capacity in a warm environment [52]. While there is some evidence of BCAA ingestion influencing ratings of perceived exertion and mental performance, the results of several well-controlled laboratory studies have failed to demonstrate a clear positive effect on exercise capacity or performance during prolonged fixed-intensity exercise to exhaustion [51], prolonged time-trial performance, incremental exercise, or intermittent shuttle running [53]. Possible reasons why BCAA supplementation does not appear to be effective could be an increase in ammonia production, as it may limit tyrosine uptake across the blood–brain barrier.

### Tyrosine

Tyrosine, or 4-hydroxyphenylalanine, can be synthesized in the body from phenylalanine, and is found in many high-protein foods such as soy products, chicken, turkey, fish, peanuts, almonds, avocados, milk, cheese, yogurt, and sesame seeds. The acute consumption of tyrosine increases the ratio of tyrosine to other large neutral amino acids such as leucine, isoleucine, valine, and tryptophan. A series of preclinical animal studies has been conducted that clearly indicate that tyrosine reduces many of the adverse effects of acute stress on cognitive performance in a wide variety of stressful environments. Studies with humans have shown that tyrosine supplementation attenuates decrements in cognitive function in sleep-deprived and chronically stressed volunteers [54, 55].

Although it has been difficult to demonstrate conclusively that tyrosine has beneficial effects in humans during exercise [50, 56, 57], partly due to ethical concerns, the majority of evidence suggests that tyrosine is useful as an acute treatment to prevent stress-related declines in cognitive function.

Exercise in heat, on the other hand, represents a specific demand on brain dopamine that is not apparent in temperate conditions [58, 59]. Therefore, the brain tyrosine requirement may be greater with the cumulative demands of exercise and heat stress, and may become limiting for dopamine synthesis and release. Tumilty et al. [60] recently assessed the effects of acute tyrosine supplementation on exercise capacity in the heat. Eight healthy male subjects cycled until exhaustion at an intensity above the lactate threshold but below the critical power threshold. That study indicated, for the first time, that supplementing a nutritional dopamine precursor 1 hour before exercise was associated with increased exercise capacity in the heat, and demonstrated that tyrosine availability, at least in part, may influence prolonged exercise tolerance with heat stress [61]. However, the authors could not reproduce the results when a simulated time trial, as the performance measure, was used [62]. Further studies are needed to identify the influence of regular supplementation of large amounts of tyrosine (5–10 g) on health due to chronic changes in sympathetic nervous system activity.

### Carbohydrate

Another nutritional strategy that may influence the development of central fatigue is carbohydrate feeding. The beneficial effect of carbohydrate supplementation during prolonged exercise could also relate to increased (or maintained) substrate delivery for the brain, with a number of studies indicating that hypoglycemia affects brain function, and cognitive performance. Carbohydrate feeding has been shown to improve higher intensity exercise performance lasting approximately 60 min, even though the estimated amount of glucose delivered to the muscle during this period was estimated to be very small [63]. In addition, no benefit of direct glucose infusion on time-trial performance [64] was found. This suggests an alternative mechanism that may involve the brain for the ergogenic effect of carbohydrate feeding during exercise. The results of a study by Dalsgaard et al. [65] indicated that glucose and lactate uptake by the brain are increased out of proportion to oxygen when the brain is activated by exhaustive exercise, and that such metabolic changes are influenced by the will to exercise. Very recently, Matsui et al. [66, 67] showed that brain glycogen could also play an important role during long-duration exercise [66] and that exercise training also creates a supercompensation of brain glycogen [67]. Those studies indicate that brain carbohydrate metabolism might also be an important factor influencing fatigue during endurance exercise.

The role of carbohydrate and a possible direct link with the brain was shown by several mouth-rinse studies. Carter et al. [68] reported a 3 % (placebo 61.37 min; carbohydrate 59.57 min) increase in performance following the rinsing of a maltodextrin solution around in the mouth before and during exercise. No solution was actually ingested during the protocol, suggesting that this performance benefit may have been mediated through direct communication between receptors present in the mouth and the brain. Other groups have also examined the effects of a carbohydrate mouth rinse on performance. Pottier et al. [69] found a performance improvement on a 60-min time trial when rinsing with a carbohydrate-electrolyte solution, while Rollo et al. [70–72] demonstrated ergogenic effects on different time trials. Interestingly, most studies that found an effect were carried out in the fasted state. When a carbohydrate mouth rinse was performed in a fed state, no effect on performance in 45-min [73] and 60-min time trials were observed [74]. The authors suggested that the oral perception of carbohydrate perhaps only plays a role when muscle and liver glycogen stores are reduced. However, this finding was not replicated in a very recent study by Fares and Kayser [75]. In that study, a mouth rinse with a maltodextrin solution increased time until exhaustion in both a fed and fasted state in non-athletic male subjects [75]. The concept of the carbohydrate mouth rinse has been supported by work investigating brain activity following the ingestion of a bolus of glucose [76], and research demonstrating the activation of several brain regions after rinsing carbohydrate solutions within the mouth [77]. Those studies highlight a marked increase in brain activation, occurring immediately after carbohydrate enters the mouth, with a second spike in activity observed 10 min following ingestion, presumably occurring as the substrate enters the circulation. These findings are novel and suggest an interesting mechanism of action. Further investigation of carbohydrate receptors in the mouth is certainly warranted.

### Caffeine

Caffeine has long been recognized as an ergogenic aid. In the past, caffeine use was restricted for athletes and it was only removed from the list of banned substances in January 2004 and added to the monitoring list. The mechanism of action of caffeine is still elusive. In the past it has been attributed to an increased availability of free fatty acids [78], resulting in a glycogen-sparing effect. However, this finding is far from conclusive, and there is now evidence that the mechanism of action of caffeine is not due to muscle glycogen sparing [79]. Current research supports a CNS effect mediated by the antagonism of adenosine receptors as the most likely cause [80]. Adenosine inhibits the release of dopamine and, therefore, caffeine induces higher brain dopamine concentrations [80]. Human studies using a variety of exercise protocols have shown performance improvements after caffeine intake [81–83]. In addition, Warren et al. [84] recently conducted a systematic review and meta-analysis of the research literature assessing the effect of caffeine ingestion on maximal voluntary contraction (MVC). They concluded that, overall, caffeine improves MVC strength and muscular endurance. Quercetin also seems to have similar effects as caffeine as it is also an adenosine receptor antagonist. However, Cheuvront et al. [85] showed that the nutritional adenosine receptor antagonists caffeine and quercetin do not enhance endurance exercise performance in the heat.

Given the widespread use of caffeine by many, the level of habitual intake may be an important factor to consider when undertaking caffeine supplementation with the view to enhancing performance. In some caffeine-naive individuals, caffeine can produce several side effects, such as tachycardia and palpitations, nervousness, dizziness, and gastrointestinal symptoms that may be detrimental to performance. These side effects can be minimized by using low doses of caffeine (e.g. 3 mg/kg body mass) as is currently recommended, while still conferring performance benefits. The positive (and possible negative) effects of caffeine seem very individually determined so previous experience with doses and timing is essential before using supplementation in competitive environments.

## Conclusion

Exercise and nutrition are both powerful means to influence the brain. The sports medicine profession are only at the start of exploring and understanding what really happens in the brain during exercise, but it is clear that physical activity and nutrition have health-enhancing effects on the brain. In the near future, nutritional interventions will also focus on brain activity during exercise.
